# Preparing and Characterizing of Xyloglucan Films Containing Tea Extract for Oral Mucositis

**DOI:** 10.1021/acsomega.4c06410

**Published:** 2024-12-19

**Authors:** Kaoru Hirose, Rieko Nitto, Shohtaro Yokota, Yayoi Kawano, Kazuhiko Yamatoya, Akira Tabuchi, Yumeo Suzuki, Takehisa Hanawa

**Affiliations:** † Faculty of Pharmaceutical Sciences, Tokyo University of Science, Noda, Chiba 2788510, Japan; ‡ MP Gokyo Food & Chemical Co., Ltd., Osaka, Kita-ku 5300001, Japan

## Abstract

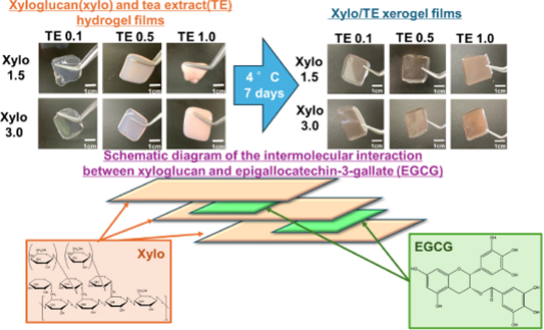

This study aimed to prepare films using Xyloglucan (Xylo) and tea extract (TE) to treat aphthous stomatitis without causing discomfort. Xylo, which gelates by adding polyphenol, was used as a gelation agent, and TE, which contains epigallocatechin-3-gallate (EGCG) with antioxidant properties, was used as an active pharmaceutical agent. Two kinds of films, hydrogel and xerogel films, were prepared by mixing various amounts of Xylo and TE. Their gelling behavior and physicochemical properties were evaluated. The sol–gel transition temperature increased with increased TE content, and the rupture strength of the films increased with increasing Xylo and TE concentrations. Rapid water absorption and high adhesiveness were observed during the reconstruction process from the xerogel to the hydrogel. The concentrations of Xylo and TE controlled the formulations’ physicochemical properties and the EGCG release rate. These results concluded that the xerogel prepared by using Xylo and TE could be applied as an oral mucosal adherent film formulation.

## 1 Introduction

Oral mucositis is an inflammatory lesion of the oral mucosa caused by the side effects of chemotherapy or radiation therapy and is often a barrier to treatment. Although the etiological factors of recurrent aphthous stomatitis (RAS) are unclear, the risk factors for RAS include smoking, bacterial or viral infections, and nutritional deprivation (iron, zinc, folic acid, or vitamin B). Furthermore, RAS is a symptom of autoimmune diseases such as Behçet’s disease, ulcerative colitis, and allergic reactions.^1,2^ Stomatitis is known to be a condition with multiple lesions, often painful, and it causes eating and sleep disturbances in patients. Significantly, painful stomatitis exacerbates the patients’ nutritional status and decreases their quality of life, delaying a patient’s cancer treatment schedule.^3,4^

Topical medicines, such as ointments, films, mouthwashes, and patches, are mainly used to treat stomatitis.^5,6^ These medicines are broadly classified into two categories: a category to prevent stomatitis through anti-inflammatory disinfectant and a category with analgesic and mucosal protection, with removing free radicals caused by chemotherapy, and therapy for stomatitis.^6,7^

Mouthwash is easy to use but requires spitting out, which may induce nausea. In addition, mouthwashes containing local anesthetics can cause discomfort in the oral cavity. Ointments applied to the oral mucosa present several problems, including reduced drug retention due to saliva and poor usability, which induce nausea during application and stickiness after application. Although mucoadhesive tablets improved drug retention in the affected area, there were new problems with usability, such as pain when applying mucoadhesive tablets and the tendency to peel off when eating, because of the tablet size and thickness.

Developing a formulation for oral mucositis that can be used easily and comfortably by patients may prevent a decline in quality of life and eating, thereby helping patients with chemotherapy and radiotherapy.

**Figure 1:**
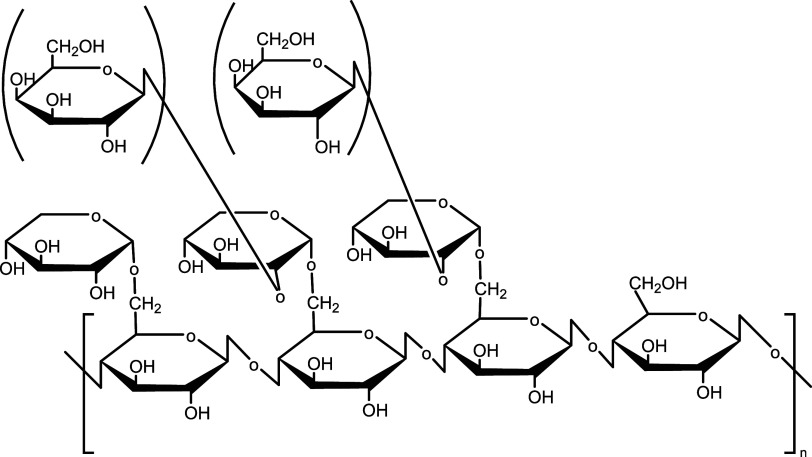
Structural formula of Xylo.

Various polymers have been used as base materials for mucoadhesive films, often containing multiple polymers such as mucoadhesive and plasticizers for films.^8−11^ Xyloglucan (Xylo) is a water-soluble polymer derived from tamarind seeds. Xylo has a β-1,4-glucan backbone chain and some α-1,6-xylose branched-side chains (Figure 1). In addition, the presence and/or absence of subunits with galactose attached to xylose side chains has been confirmed, and the repeating unit consists mainly of four types of oligosaccharides.^12−14^

Xyloglucan (Xylo) has been used as a food thickener, stabilizer, and gelling agent in Japan, Taiwan, Korea, China, the USA, and other Asian countries.^14^ Xylo has undergone a battery of rigorous safety studies, including acute, subacute, mutagenic, and chronic toxicity studies. There has been no report of TSX-related abnormal findings through all of these studies.^14^ The solution exhibits typical Newtonian flow properties similar to starch and is very stable in heat, pH, and shear. They added large amounts of sugar, alcohol,^15^ polyphenols, and dyes such as Congo Red, resulting in Xylo gelation. According to Yuguchi et al., gelation occurs when the cellulose-like backbone of Xylo aggregates and laminates in parallel through gelling agents, and the interaction between Xylo and the gelling agents is thought to involve van der Waals forces or hydrogen bonds.^16^

Furthermore, Xylo gel forms a molecular structure similar to the mucin network in the oral mucosa and has mucoadhesive properties.^17^ Although there have been reports of preparations of Xylo gel, the targets of these formulations are the gastrointestinal tract, cornea, and nasal mucosa.^18−20^ However, there have been few reports of their use in treating stomatitis.

**Figure 2:**
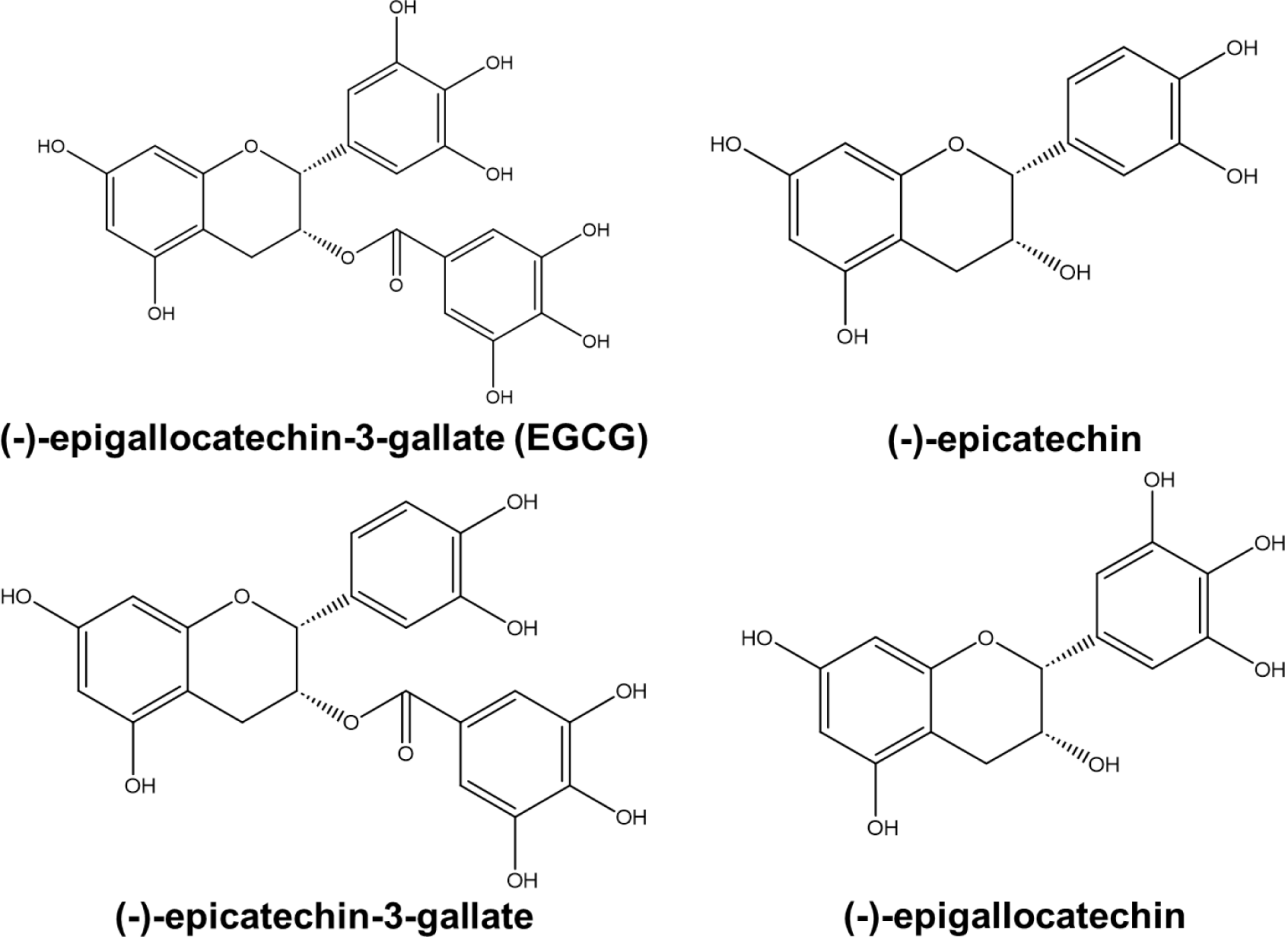
Structural formula of leading catechins in green tea extract.

Additionally, epigallocatechin-3-gallate (EGCG)-induced gelation of Xylo has been reported.^21,22^ EGCG (Figure 2), a derivative of catechin extracted from tea leaves, has been reported to have antioxidant, antitumor, and antibacterial effects and to inhibit biofilms.^23,24^ The application of EGCG in treating of oral mucositis is currently being investigated, both in developing of novel formulations^25^ and within a medical practice front.^26^

Although gels consisting of Xylo and EGCG have been reported as food additives,^18^ there are no examples of their application in formulations.

Therefore, this study focused on the availability of mucoadhesive films to improve the usability of topical oral medications. Mucoadhesive films are lightweight, thin, and portable, and can overcome the poor usability of mucoadhesive tablets.^27,28^ In this study, mucoadhesive films composed of Xylo and tea extracts from green tea (TE) (containing catechin >75% and EGCG >40%) were prepared to prevent and heal oral stomatitis. Given the availability of TE containing not only EGCG but also a variety of chatechin^29^ (Figure 2) at a low cost in Japan, this TE was elected to utilize this tea extract as a gelling agent for xyloglucan in the present study. Before being applied to clinical use, the gelation behaviors of Xylo and TE and the physicochemical properties of the gels and films were investigated.

## 2 Materials and Methods

### 2.1 Materials

Xylo was supplied by MP Gokyo Food & Chemical Co. (Osaka, Japan). The TE Sunphenon 90MB-OP (containing >75% catechin and >40% EGCG) was provided by Taiyo Kagaku Co. (Tokyo, Japan). Mucin from the stomachs of pigs was purchased from Sigma-Aldrich Co., LLC. (St. Louis, MO, USA). Carmellose sodium (CMC-Na), KCl, CaCl_2_, MgCl_2_, and K_2_HPO_4_ were purchased from Kanto Chemical Co., Inc. (Tokyo, Japan). D-sorbitol and NaCl were purchased from Wako Pure Chemical Industries, Ltd. (Osaka, Japan). EGCG was purchased from Struchem Co. Ltd. (JiangSu, China). All chemicals and solvents used were of analytical grade.

### 2.2 Experimental Methods

#### 2.2.1 Preparation of the Xylo/TE Hydrogel and Xerogel Film

Xylo solutions (1.5 or 3.0%, w/w) and TE solutions (0.1, 0.5, and 1.0%, w/w) were prepared with ultrapure water. The TE solution was added to the Xylo solution and stirred using a three-one motor (FBL 600; Shinto Scientific. Co., Ltd., Tokyo, Japan). The Xylo/TE solutions were dispensed into 2 × 2 cm^2^ balance dishes. The solutions were allowed to stand for 24 h at 4 °C, and hydrogel films were prepared. Furthermore, the Xylo/TE xerogel film was prepared by allowing the Xylo/TE hydrogel films to stand at 4 °C for 7 days.

#### 2.2.2 Infrared Absorption Spectroscopy

Fourier-transform infrared (FT-IR) spectroscopy was performed to determine the molecular interactions between Xylo and TE. Physical mixtures (PM) of Xylo and TE were prepared using a vortex mixer (VORTEX-GENIE 2, M&S Instruments Inc., Osaka, Japan) and mixed for 1 min. Infrared spectra were obtained using a Frontier FT-IR spectrometer (PerkinElmer Japan G.K., Kanagawa, Japan) at 25 °C in the 4000–400 cm^–1^ range and cumulated 16 times.

#### 2.2.3 Measurement of the Sol–Gel Transition Temperature of Xylo/TE Hydrogel

**Figure 3:**
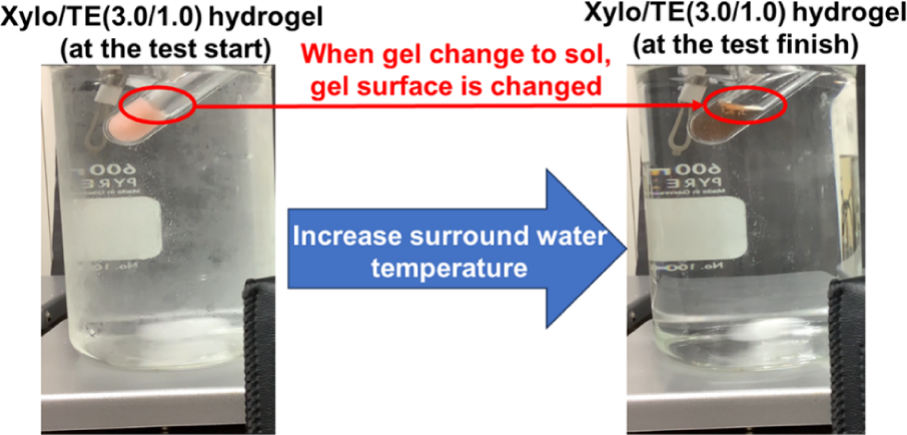
Evaluation of the sol–gel transition of the Xylo/TE hydrogels.

A modified inversion test was used to investigate the sol–gel transition temperature. The test tube containing the gel was fixed in a beaker with water at 45° tilt. The water temperature was increased from 4 to 50 °C by stirring the water (650 rpm) on a hot plate. The temperature at which the change in the gel surface was observed was defined as the sol–gel transition temperature (Figure 3).

#### 2.2.4 Water Content and Volume Change for Xylo/TE Hydrogel at Storage

Hydrogel films (φ15 mm) were stored in specific conditions (35 °C, 28% RH) at 24 h, and water content and volume change rates were measured at definite intervals: 0, 0.25, 0.5, 0.75, 1, 1.5, 2–8 (every hour), and 24 h. The water content rate and volume change were calculated using eqs 1 and 2, respectively. Vernier calipers were used to measure the film volume.


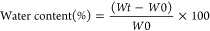
1
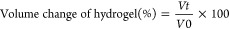
2

*W*_0_ is the initial weight (mg), *W*_*t*_ is the weight of gel (mg),

*V*_0_ is the initial volume (mm^3^), and *V*_*t*_ is the gel volume (mm^3^).

#### 2.2.5 Rupture Strength of Xylo/TE Hydrogel and Xerogel Films

The mechanical strength of the hydrogel and xerogel films was measured immediately after preparation except that the xerogel films were allowed to absorb water for 20 min before the measurement. The film was fixed to the sample stage of a creep meter (RHEONER II, RE2–3305C; Yamaden Co., Ltd., Tokyo, Japan). A plunger (φ = 1 mm) was brought into contact with the film surface and displaced perpendicularly to the film surface at a speed of 5 mm/s until the film was perforated. The load that the plunger received from the film and the distance of the plunger from its original position were recorded. The maximum load in the stress–strain curve is generated when the film ruptures, which is defined as the rupture strength.

#### F2.2.6 Water Absorption Behavior of Xylo/TE Xerogel Films

A circularly cut xerogel film (15 mm diameter) was immersed in ultrapure water (20 mL), and the weight of the film was measured at regular intervals. The water-absorption rate was calculated using eq 3.
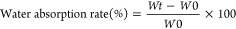
3

*W_t_* is the weight of the xerogel film after water absorption (mg), and and *W*_0_ is the initial weight of the xerogel film (mg).

#### 2.2.7 Evaluation of Xylo/TE Xerogel Film Adhesion and Comparison with Over-The-Counter (OTC) Medicines

**Figure 4:**
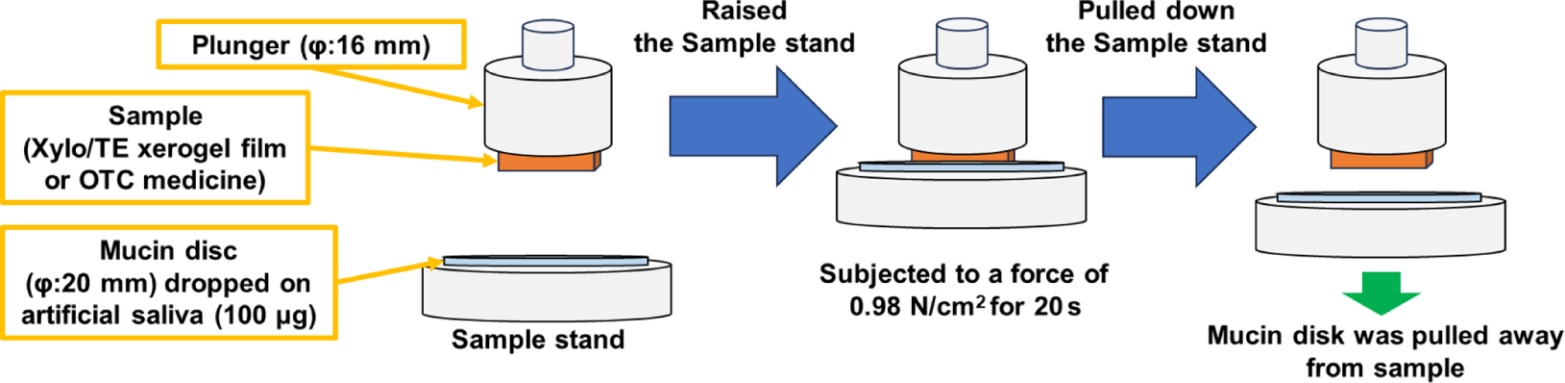
Schematic diagram of the adhesion test on a mucin disk.

Mucin disks and artificial saliva were prepared to mimic the oral mucosa. Mucin disks were prepared by dropping a 10% mucin solution (2 mL) onto filter paper (55 mm in diameter) and dried at room temperature (25 ± 1 °C) for 12 h. Artificial saliva was prepared using CMC-Na, D-sorbitol, KCl, NaCl, CaCl_2_, MgCl_2_, and K_2_HPO_4_based on the composition of pharmaceutical product (Saliveht Aerosol) used for xerostomia in Japan.^30^ A creep meter (RHEONER II, RE2–3305C; Yamaden Co., Ltd., Tokyo, Japan) was used to measure the adhesion force of the xerogel films and the use of OTC medicines for oral mucositis. The xerogel film or the OTC medicine (1 × 1 cm^2^) was attached to a plunger (16 mm in diameter), and mucin disks (20 mm in diameter) were attached to the movable sample stand. After 100 μL of artificial saliva was dropped onto the mucin disk for 60 s, the mucin disk and the sample were subjected to a force of 0.98 N/cm^2^ for 20 s (Figure 4). Adhesion force was the tension when the mucin disk was pulled away from the xerogels or the OTC medicines.

#### 2.2.8 Evaluation of EGCG Release Behavior from Xylo/TE Xerogel Films

While the xerogel films were placed in 200 mL of purified water and stirred with a magnetic stirrer at 100 rpm, samples were taken for analysis at definite intervals. The amount of EGCG released from the films was evaluated in the mobile phase composed of water for HPLC: methanol: 0.2 mol/L phosphate buffer (pH 3.0) (33:12:5) using a Shodex C18M4D (4.6 mm × 150 mm, column temperature: 40 °C) and high-performance liquid chromatography (HPLC, JASCO Co., Ltd., Tokyo, Japan) with UV detection (UV-2075 Plus; JASCO Co., Ltd., Tokyo, Japan) at 270 nm, at a flow rate of 1.0 mL/min. The retention time of EGCG is 6.3 min. To evaluate the degradability of EGCG, the dissolution of EGCG alone was measured under similar conditions.

#### 2.2.9 Statistical Analysis

The results were expressed as the mean ± standard deviation (S.D.). Statistical analysis was performed on all data except the sol–gel transition temperature using JMP Pro 17.2.0. The differences between groups were compared using the Tukey-Kramer method to determine statistical differences except for the EGCG dissolve test from the Xerogel films. The EGCG released from xerogel films was compared using Dunnett’s test (vs EGCG alone). The results of statistical analysis (rupture strength and adhesion force) are presented in the Supporting Information (*p < 0.05, **p < 0.01).

## 3 Results and Discussion

### 3.1 Appearance of Xylo/TE Gel Films

**Figure 5:**
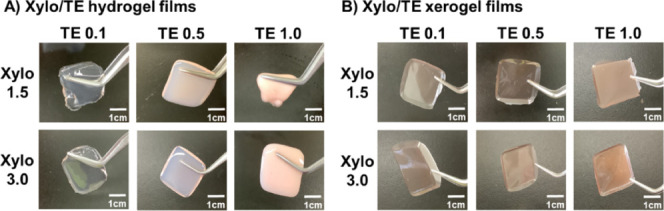
Photographs of Xylo/TE hydrogel films and their xerogel films. (A) Xylo/TE hydrogel films and(B) Xylo/TE xerogel films at room temperature.

The obtained Xylo/TE hydrogel films were slightly brown, and their transparency decreased as the TE content increased. The xerogel film became a highly transparent brown film-like substance while maintaining its planar structure (Figure 5).

### 3.2 FT-IR Spectra of Xylo/TE Xerogel Films

**Figure 6:**
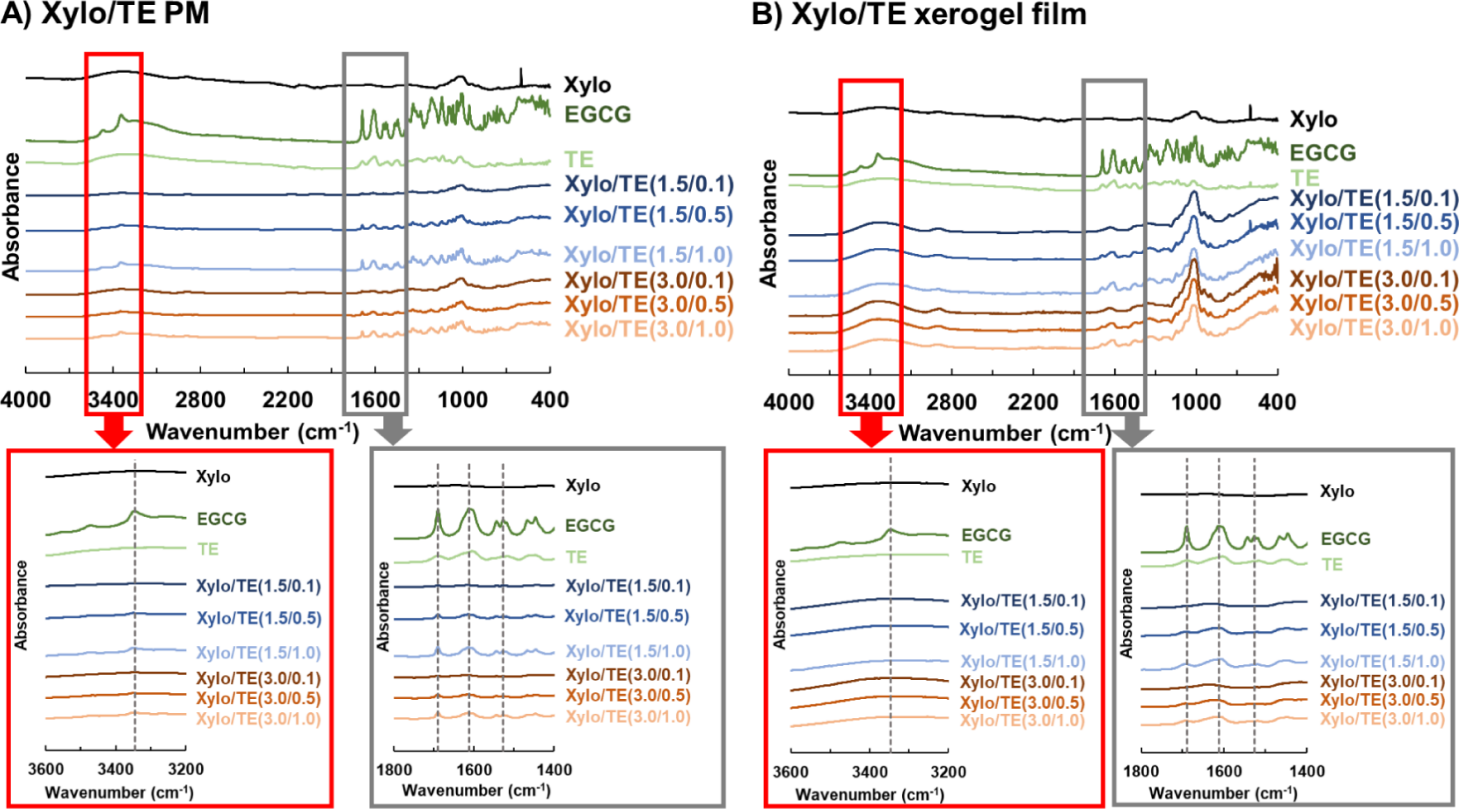
FT-IR spectra of Xylo, TE, and EGCG and their physical mixtures and xerogel films. Xylo, TE, EGCG and (A) their physical mixtures, and (B) their xerogel films. Vertical axis: absorbance, horizontal axis: wavenumber (cm^–1^). Upper diagram: the wavenumber range is 400–4000 cm^–1^, under diagram: the wavenumber range is 1400–1800 cm^–1^ (gray frame). A signal corresponding to the benzene ring appeared. The wavenumber range is 3200–3600 cm^–1^ (red frame), with a signal corresponding to the hydroxy group.

The FT-IR spectra of PM are shown in Figure 6A, and the Xylo/TE xerogels are shown in Figure 6B. Signals corresponding to the benzene rings in EGCG were observed at 1527 cm^–1^, 1613 cm^–1^, and 1690 cm^–1^, which are characteristics of EGCG. The peak intensities of PM decreased as the TE content decreased. However, no peak shift was observed; there were no intermolecular interactions between Xylo and TE. Similar results were observed for xerogels (Figure 6B). The peak at around 3350 cm^–1^, attributed to the hydroxy group, showed a decrease in peak intensity in the PM, depending on the reduction in the TE content. This peak almost disappeared for the xerogel. Among some reported gelation mechanisms of Xylo, one has been recognized as an interaction between the molecular chains of Xylo and molecules with planar structures 18. The benzene ring of EGCG was not observed for Xylo/TE xerogel films because of the insertion of EGCG between the linear chains of Xylo. It has also been suggested that the Xylo/TE hydrogel film retains the same structure as the xerogel film after water desorption.

### 3.3 The Sol–Gel Transition Temperature of Xylo/TE Hydrogel

**Figure 7:**
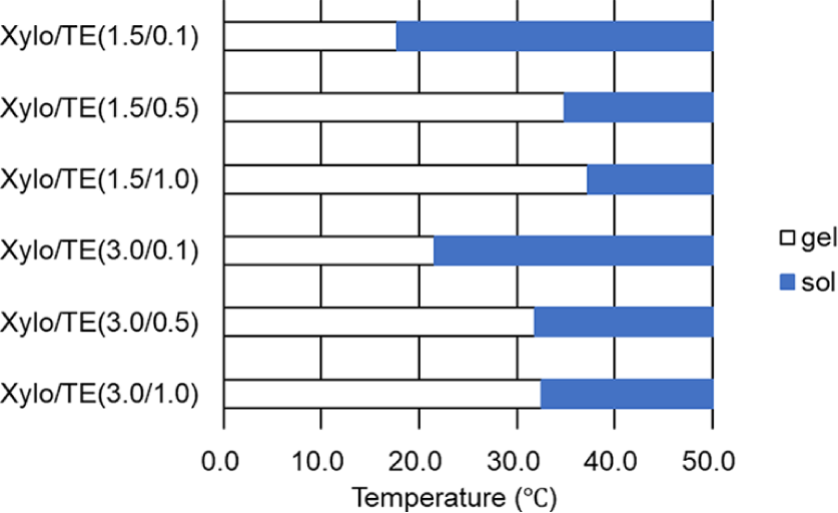
Sol–gel transition temperature of Xylo/TE hydrogels.

Retaining the shape of the formulation in the oral cavity influences the drug release behavior. The transition temperatures from gel to sol were investigated because the prepared Xylo/TE hydrogel film may dissolve because of saliva and the oral temperature when applied. All gels transitioned to sol as the surrounding temperature increased from room temperature (approximately 20 °C) to the skin surface temperature (35–37 °C) (Figure 7). As for the gel dissolution temperature, Nitta et al. demonstrated that from the rheological and DSC measurements, the gel dissolution temperature increased with an increase of the mixing weight ratio of EGCG, ranging from 30 to 40 °C.^21^ The dissolution temperature of the gels observed in this study was generally consistent with that reported by Nitta et al.^21^

These results suggest that Xylo/TE hydrogels may not maintain their formulation shape and dissolve in the oral cavity.

Therefore, further investigation of the formulation design is necessary to increase the transition temperature from the gel to the sol and improve the retention of the formulation in the mouth. This will prolong the release time of TE from the film applied to the oral mucosa, and it is thought that the healing effect of oral mucositis will be sustained.

### 3.4 Water Desorption Behavior of Xylo/TE Hydrogel Films in Storage

Immediately after preparation, the hydrogel film contained 95–98% water by weight: [Xylo/TE(1.5/0.1)], 98.1 ± 0.10%; [Xylo/TE(1.5/0.5)], 97.3 ± 0.05%; [Xylo/TE(1.5/1.0)], 96.9 ± 0.05%; [Xylo/TE(3.0/0.1)], 96.4 ± 0.10%; [Xylo/TE(3.0/0.5)], 95.9 ± 0.04%; and [Xylo/TE(3.0/1.0)], 95.2 ± 0.12%.

**Figure 8:**
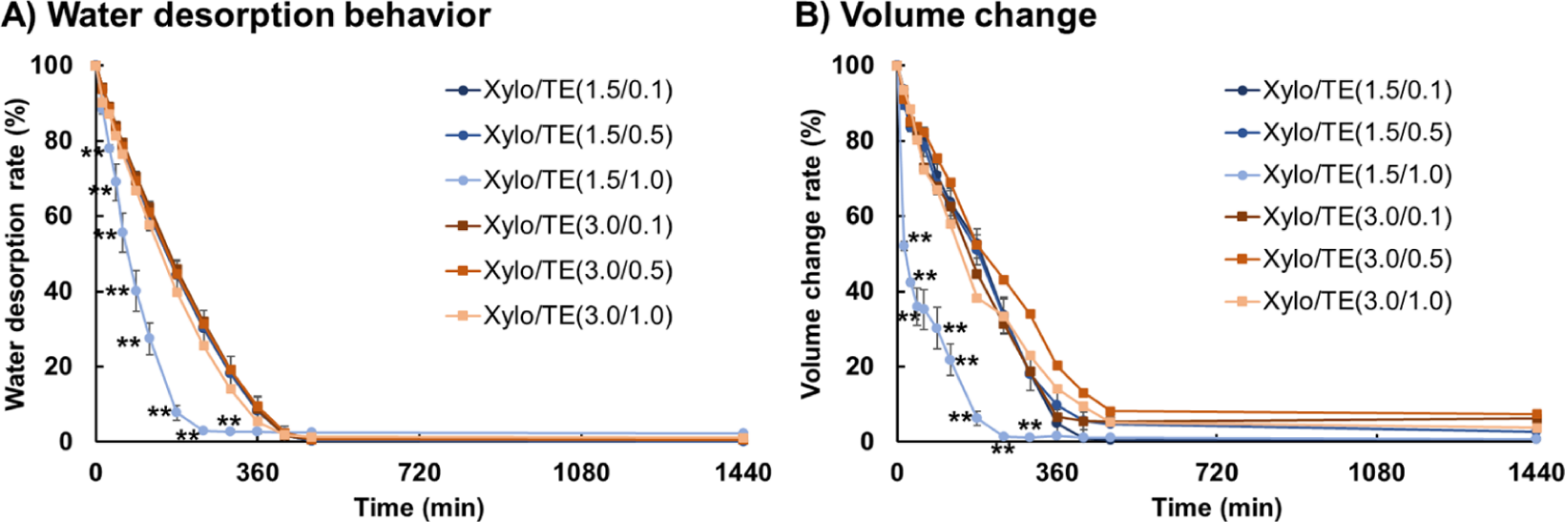
Water desorption behavior and volume change of the Xylo/TE hydrogel films. (A) Water desorption behavior from Xylo/TE hydrogel films and(B) volume change of Xylo/TE hydrogel films. The results are given as the mean ± SD (*n* = 3), **: *p* < 0.01, Xylo/TE(1.5/1.0) vs another sample.

Figure 8 shows the water desorption behavior (Figure 8A) and volume change (Figure 8B) of the hydrogels stored under 35 °C, 28% RH. Shrinkage and volume reduction were observed for all films but did not dissolve during the measurement. Thus, [Xylo/TE (1.5/1.0)] hydrogel films desorbed water and decreased in volume at an early stage. The other formulations showed no significant differences; more than 50% of the water was released from the hydrogel after 180 min and more than 90% after 360 min. The hydrogel volume decreased to less than 10% after 480 min.

The volume change and water release from the Xylo/TE hydrogel film were assumed to be due to water evaporation from the surface of the hydrogel film, based on changes in the hydrogel film’s shape over time. Compared to the other formulations, the rapid water release from the [Xylo/TE (1.5/1.0)] hydrogel films indicates a difference in the water retention capacity within the gel, presumably promoting water release by adding an excess amount of TE relative to Xylo.

### 3.5 Rupture Strength Test of Xylo/TE Hydrogel and Xerogel Films

**Figure 9:**
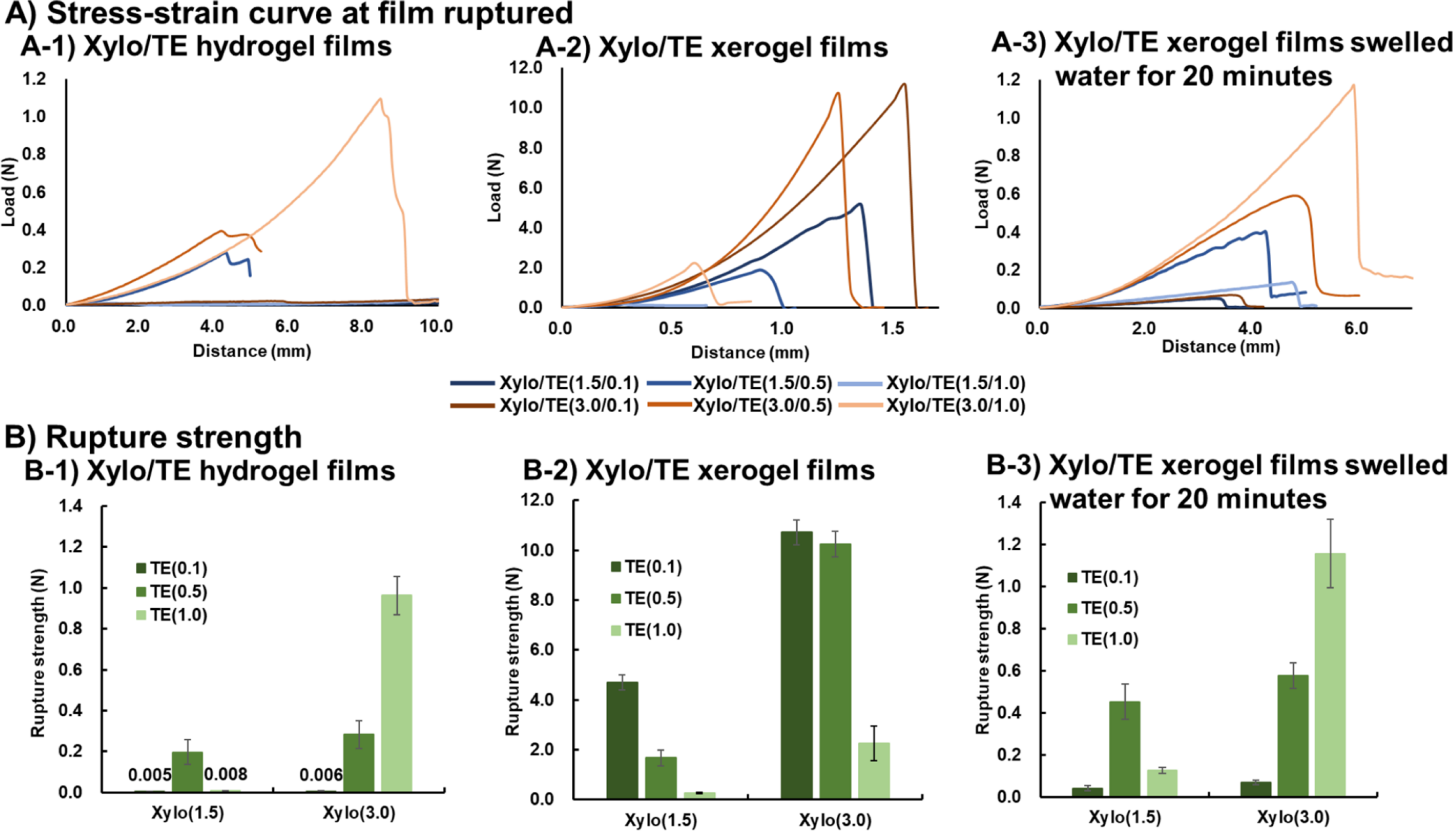
Mechanical properties of the Xylo/TE gel films. (A) Stress–strain curves at Xylo/TE gel film rupture and (B) Rupture strength of Xylo/TE gel films. The results are given as the mean ± SD in rupture strength (*n* = 3). The results of the statistical analysis are presented in Tables S1–S3.

Figure 9 shows the rupture strengths of the Xylo/TE films. Figure 9A shows a typical stress–strain curve for each film. The displacement of the plunger from its original position until the film ruptures is related to the deformation (extension) of the film, indicating that the longer the distance, the higher the film’s extensibility.

The hydrogel films showed relatively low rupture strengths compared to [Xylo/TE (3.0/1.0)] and could be challenging to handle. In the [Xylo (3.0)] hydrogel films, it was observed that the increased TE content was associated with increased rupture strength. The [Xylo/TE (3.0/1.0)] hydrogel film had the highest rupture strength (ca. 1.0 N) and extensibility compared to other hydrogel films. Among the [Xylo (1.5)] hydrogel films, the highest rupture strength was obtained for [TE (0.5)]. The rupture strength of the Xylo/TE hydrogel films varied depending on the ratio of Xylo to TE; hydrogel films containing [Xylo/TE (3.0/1.0)] and [Xylo/TE (1.5/0.5)] had a 3:1 weight ratio of Xylo to TE indicating the specific stoichiometry of these components for the mechanical strength of the hydrogel films.

Nevertheless, the results obtained with xerogel films differed from those observed with the hydrogel films. The rupture strength of the xerogels decreased as the TE concentration increased, regardless of the Xylo concentration. The main difference between the xerogel film and hydrogel film is the presence or absence of water. The structure of the hydrogel film is influenced by the interactions between Xylo and EGCG, which form a complex like an interaction complex due to the hydrophobic and/or hydrophilic interaction between Xylo and EGCG. Meanwhile, the xerogel films contain less water than hydrogel films, reducing hydrogen bonding by water molecules.

The decrease in rupture strength that accompanies the increase in EGCG content in xerogel films suggests that EGCG penetrates between the xyloglucan molecular chains and interferes with the formation of robust gels due to the formation of hydrophobic bonds between the Xylo molecular chains. However, the exact ratio of each bond in the xyloglucan/TE gel film structure remains unknown, and further investigation is needed.

Comparing hydrogel and xerogel films, xerogel films showed higher rupture strength but lower extensibility than hydrogel films. Therefore, to study the reversibility of the xerogel film, the rupture strength of the swollen xerogel film owing to water absorption for 20 min was examined. The rupture strength of the swollen xerogel film due to water absorption was higher than that of the hydrogel film, and the distance was similar to that of hydrogel films. Accordingly, the xerogel films were considered to reversibly transit to a hydrogel film state by water absorption.

### 3.6 Water Absorption Behavior of Xylo/TE Xerogel Films

**Figure 10:**
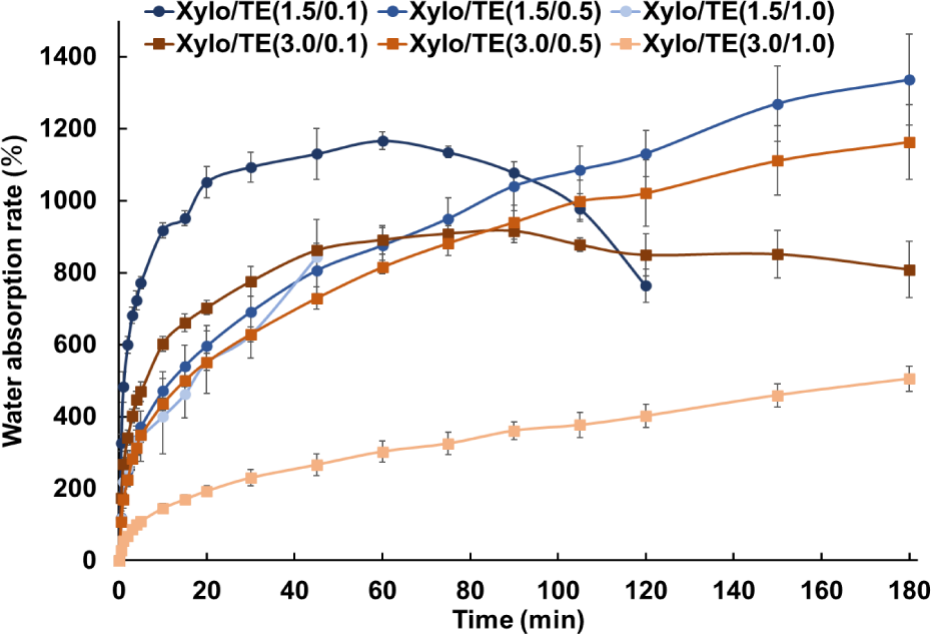
Water absorption behavior in Xylo/TE xerogel films. The results are given as the mean ± SD (*n* = 3). The results of the statistical analysis, which show the xerogel films that had been absorbing water for 30 min, are presented in Table S4.

The water absorption rate up to 30 min after the start of the test tended to decrease as the Xylo and TE contents increased (Figure 10). The xerogel films, [Xylo/TE (1.5/0.1)] and [Xylo/TE (1.5/1.0)], disintegrated during tests completely; each disintegration time was at 120 and 45 min, respectively. The [Xylo (3.0)] xerogel films maintained their shape throughout the tests. The [TE (0.5)] group and [Xylo/TE (3.0/1.0)] formulations exhibited sustained water absorption. The reduction in water absorption at [Xylo/TE (1.5/0.1)] and [Xylo/TE (3.0/0.1)] was assumed to be due to the evaporation of water from the surface, or the disintegration of hydrogel might occur. In particular, the [Xylo/TE (1.5/0.1)] exhibits the least amount of gel material for both Xylo and TE. Therefore, it could be considered that the water retention reaches its limit at 60 min, resulting in the release of water and the disintegration of the gel.

### 3.7 Evaluation of Adhesiveness of Xylo/TE Xerogel Films

**Figure 11:**
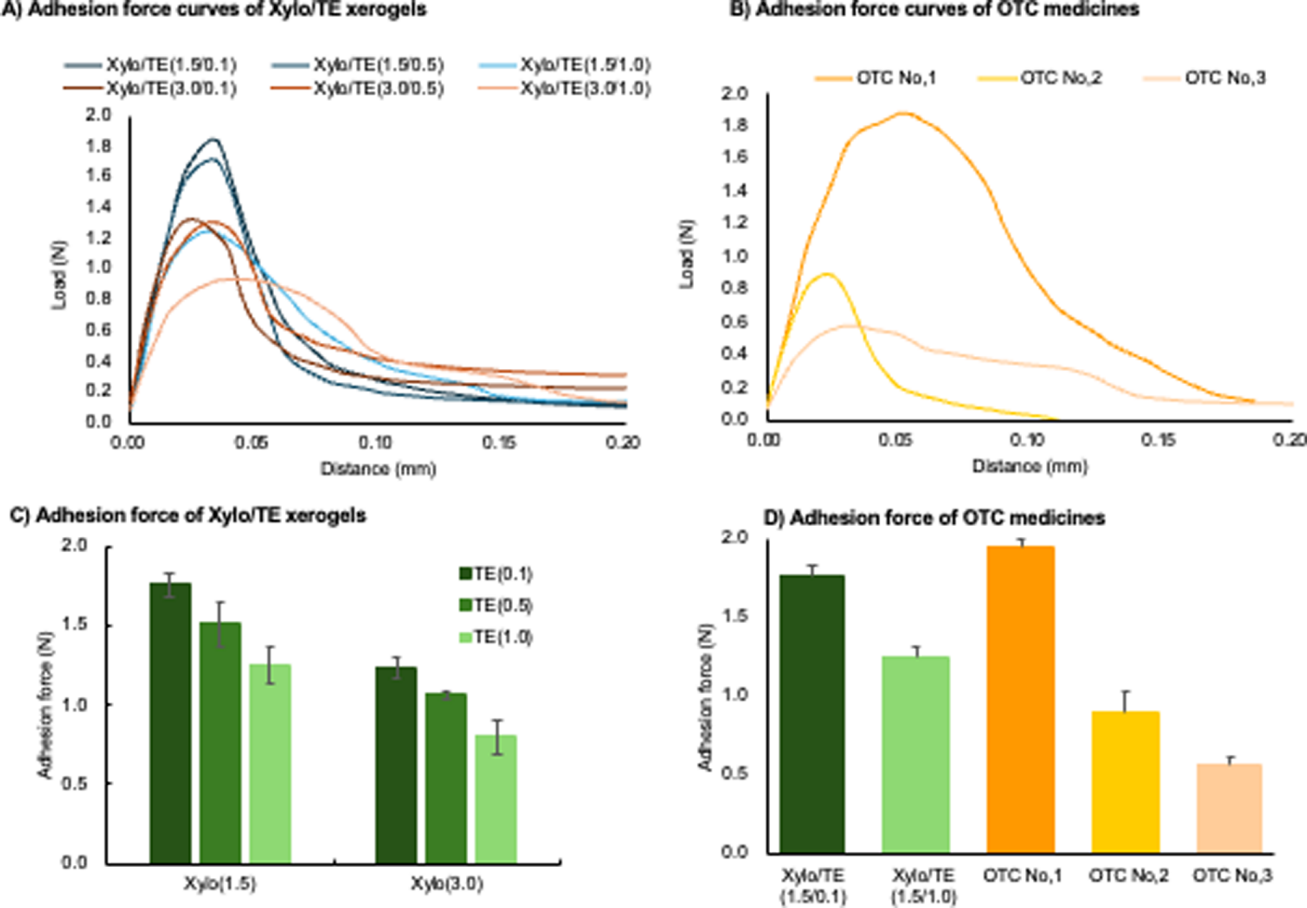
Adhesion force curves and adhesion of Xylo/TE xerogel films and commercial films. (A) Adhesion force curves of Xylo/TE xerogel films,(B) adhesion force curves of commercial films,(C) adhesion force of Xylo/TE xerogel films,and (D) adhesion force of commercial films and Xylo/TE xerogel films. The results are the mean ± SD in parts (C) and (D)(*n* = 3). The results of the statistical analysis are presented in Table S5.

To evaluate the adhesiveness of the hydrogels to the oral mucosa, mucin disk and artificial saliva were used as in vitro models that mimicked the oral cavity (Figure 11). Figure 11A,B presents the stress change produced until the mucin disk and xerogel films were brought into contact for 20 s and then separated. The maximum stress of the curve represents the stress just before the xerogel film peels off from the mucin disk, which is defined as the adhesion force in this study. The distance between the mucin disk and xerogel film when the xerogel film peeled off was similar to all Xylo/TE groups; however, the adhesion force decreased as the amount of TE in the hydrogel increased.

The adhesion of polymers to mucins is an intricate phenomenon influenced by many factors, including the polymer’s structural characteristics, the solution’s viscosity, and contact time with mucin.^31^ One of the mechanisms observed is the hydration interaction between mucins and polymers, which is driven by the movement of water molecules between mucins and polymers.^32^ The xerogel film that showed high adhesion to mucin disks was a formulation containing [TE (0.1)], which was thought to be related to faster water absorption (Figure 10). The [TE (0.1)] formulation demonstrated the highest degree of adhesion. It exhibited the quickest rate of water absorption during the water absorption test. Therefore, it was assumed that the high adhesion of [TE (0.1)] was due to rapid water absorption from the mucin disk.

The adhesion of the OTC oral mucoadhesive films sold in Japan was compared with that of the Xylo/TE xerogel films. [Xylo/TE (1.5/0.1)] and [Xylo/TE (3.0/0.1)], which have high adhesion, showed adhesive forces equivalent to those of the OTC medicines. The adhesives used in the OTC medicines in this study are listed in Table 1. Adhesion to mucin differs depending on the adhesive, and it is known that adhesion strength increases in the order of hydroxy methyl polymer, carboxy vinyl polymer, and Xylo.^33^ This report suggests that Xylo/TE xerogel films have an adhesion strength equal to or greater than that of the OTC medicines. This indicates the potential for high adhesion to the oral mucosa without adhesives.

**Table 1:** Excipients of the OTC Mucosal Adherent Film for Aphthous Oral Mucositis

OTC No.	Excipients	Adhesive compound
No. 1	Povidone, hydroxypropyl cellulose, tannic acid, citric acid, polyethylene glycol, titanic oxide, ethyl cellulose, coloring	
No. 2	Polyacrylic acid, triethyl citrate, hydroxypropylmethyl cellulose, ethyl cellulose, castor oil, titanic oxide	Hydroxypropylmethyl cellulose
No. 3	Hydroxypropyl cellulose, caboxyvinyl polymer, magnesium stearate, lactose, carboxymethyl cellulose, talc, magnesium aluminometasilicate, coloring	Carboxyvinyl polymer

### 3.8 Measurement of Dissolution of EGCG from Xylo/TE Xerogel Films

**Figure 12:**
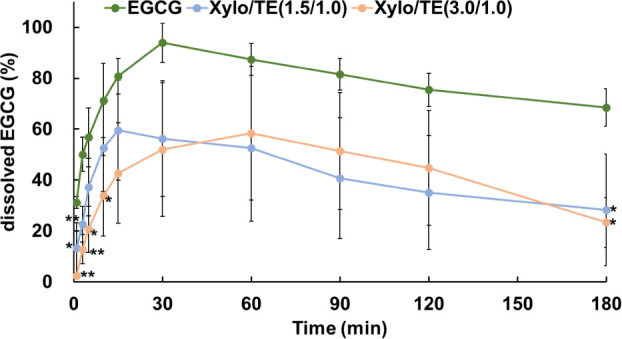
Release profiles of EGCG from Xylo/TE xerogel films containing TE 1.0%. The results are given as the mean ± SD (*n* = 3), *: *p* < 0.05, **: *p* < 0.01, vs EGCG.

The results of EGCG dissolution from the Xylo/TE xerogel films containing [TE (1.0)] are shown in Figure 12. As a control, EGCG pure powder was used.

The highest concentrations of EGCG released from each formulation were 59.7% (the cumulative release weight was 5.10 mg) for [Xylo (1.5)] and 58.4% (the cumulative release weight was 4.95 mg) for [Xylo (3.0)]. The time required to achieve the maximum released concentration of EGCG has tended to increase with increasing Xylo concentration ([Xylo (1.5)]: 15 min; [Xylo (3.0)]: 60 min). In both formulations, the EGCG concentration decreased 60 min after the dissolution test, suggesting that EGCG degradation may have occurred over time during the test. To verify the degradation of EGCG, concentration changes in the EGCG solution were measured under the same conditions. The TE solution showed a decrease in the EGCG concentration 60 min after the start of the test. Tea catechins have been reported to be degraded by various factors, including oxidation, temperature, and pH.^34,35^ Because dissolution was performed at 37 °C and the HPLC column temperature was set to 40 °C in this test, it was speculated that TE released from the xerogels may have degraded over time.

Furthermore, although the functional activity of the EGCG that was released was not evaluated in this study as HPLC measured stable EGCG, it is thought to be stable at the time of release. However, the long-term storage stability needs to be evaluated in the future.

## 4 Conclusion

These findings suggest that varying the amount of xyloglucan and catechin can regulate the gel strength, adhesion, water absorption, and dissolution rate of EGCG. Xylo/TE xerogel films exhibited high strength, and their water absorption enabled them to achieve properties similar to those of hydrogel films. Furthermore, in the adhesion test, Xylo/TE xerogel films presented an equivalent adhesion force to commercial films.

Despite the identification of several issues in this study, the xerogel film prepared with xyloglucan and tea extract has the potential to be applied as an oral mucosa-adhesive film. In future studies, improvement of the Xylo/TE gel film will be considered to refine the formulation’s physical properties and to evaluate its safety and efficacy as a treatment for oral mucositis using cells experiments.

## Supporting Information Available

The results of statistical analysis in rupture strength, adhesion force, and water absorption at 30 min (PDF)

## Abbreviations

Xylo: xyloglucan
TE: tea extract
EGCG: epigallocatechin-3-gallate
RAS: recurrent aphthous stomatitis
FT-IR: Fourier-transform infrared
PM: physical mixture
OTC: over-the-counter
HPLC: high-performance liquid chromatography

## References

[ref1] Chavan M.; Jain H.; Diwan N.; Khedkar S.; Shete A.; Durkar S. Recurrent aphthous stomatitis: A review. J. Oral Pathol. Med. 2012, 41 (8), 577–583. 10.1111/j.1600-0714.2012.01134.x. 22413800

[ref2] Porter S. R.; Scully C.; Pedersen A. Recurrent aphthous stomatitis. Crit. Rev. Oral Biol. Med. 1998, 9 (3), 306–321. 10.1177/10454411980090030401. 9715368

[ref3] Mead G. M. Management of oral mucositis associated with cancer chemotherapy. Lancet 2002, 359 (9309), 815–816. 10.1016/S0140-6736(02)07960-6. 11897276

[ref4] Lalla R. V.; Bowen J.; Barasch A.; Elting L.; Epstein J.; Keefe D. M.; McGuire D. B.; Migliorati C.; Nicolatou-Galitis O.; Peterson D. E.; et al. MASCC/ISOO clinical practice guidelines for the management of mucositis secondary to cancer therapy. Cancer 2014, 120 (10), 1453–1461. 10.1002/cncr.28592. 24615748 PMC4164022

[ref5] Yasu T.; Momo K.; Horii M.; Yokoyama K.; Ono K.; Kiyomi A.; Sugiura M.; Kuroda S. Effect of Indomethacin Mouthwash on Pain Due to Chemotherapy-Induced Oral Mucositis. J. Palliat. Med. 2020, 23 (7), 886–887. 10.1089/jpm.2020.0116. 32609605

[ref6] Suharyani I.; Fouad Abdelwahab Mohammed A.; Muchtaridi M.; Wathoni N.; Abdassah M. Evolution of Drug Delivery Systems for Recurrent Aphthous Stomatitis. Drug Des., Dev. Ther. 2021, 15, 4071–4089. 10.2147/DDDT.S328371. PMC8489189 34616142

[ref7] Tagami T.; Okamura M.; Ogawa K.; Ozeki T. Fabrication of Mucoadhesive Films Containing Pharmaceutical Ionic Liquid and Eudragit Polymer Using Pressure-Assisted Microsyringe-Type 3D Printer for Treating Oral Mucositis. Pharmaceutics 2022, 14 (9), 1930 10.3390/pharmaceutics14091930. 36145678 PMC9505851

[ref8] Shipp L.; Liu F.; Kerai-Varsani L.; Okwuosa T. C. Buccal films: A review of therapeutic opportunities, formulations & relevant evaluation approaches. J. Controlled Release 2022, 352, 1071–1092. 10.1016/j.jconrel.2022.10.058. 36351519

[ref9] Alves T. F. R.; Rios A. C.; da Silva Pontes K.; Portella D. L.; Aranha N.; Severino P.; Souto E. B.; Gonsalves J. K. M.; de Souza Nunes R.; Chaud M. V. Bilayer Mucoadhesive Buccal Film for Mucosal Ulcers Treatment: Development, Characterization, and Single Study Case. Pharmaceutics 2020, 12 (7), 657 10.3390/pharmaceutics12070657. 32664574 PMC7408552

[ref10] Milanda T.; Cindana Mo’o F. R.; Mohammed A. F. A.; Elamin K. M.; Wilar G.; Suharyani I.; Wathoni N. Alginate/Chitosan-Based Hydrogel Film Containing α-Mangostin for Recurrent Aphthous Stomatitis Therapy in Rats. Pharmaceutics 2022, 14 (8), 1709 10.3390/pharmaceutics14081709. 36015335 PMC9414115

[ref11] Salamat-Miller N.; Chittchang M.; Johnston T. P. The use of mucoadhesive polymers in buccal drug delivery. Adv. Drug Delivery Rev. 2005, 57 (11), 1666–1691. 10.1016/j.addr.2005.07.003. 16183164

[ref12] Gidley M. J.; Lillford P. J.; Rowlands D. W.; Lang P.; Dentini M.; Crescenzi V.; Edwards M.; Fanutti C.; Reid J. S. Structure and solution properties of tamarind-seed polysaccharide. Carbohydr. Res. 1991, 214 (2), 299–314. 10.1016/0008-6215(91)80037-n. 1769022

[ref13] York W. S.; van Halbeek H.; Darvill A. G.; Albersheim P. Structural analysis of xyloglucan oligosaccharides by 1H-n.m.r. spectroscopy and fast-atom-bombardment mass spectrometry. Carbohydr. Res. 1990, 200, 9–31. 10.1016/0008-6215(90)84179-X. 2379217

[ref14] Nishinari K.; Takemasa M.; Suzuki Y.; Yamatoya K.; Phillips G. O.; Williams P. A. 11 - Xyloglucan. In Handbook of Hydrocolloids; 3rd ed.; Woodhead Publishing, 2020, pp. 317–365.

[ref15] Yamanaka S.; Yuguchi Y.; Urakawa H.; Kajiwara K.; Shirakawa M.; Yamatoya K. Gelation of tamarind seed polysaccharide xyloglucan in the presence of ethanol. Food Hydrocolloids 2000, 14 (2), 125–128. 10.1016/S0268-005X(99)00057-0.

[ref16] Yuguchi Y.; Hirotsu T.; Hosokawa J. Structural characteristics of xyloglucan - Congo red aggregates as observed by small angle X-ray scattering. Cellulose 2005, 12 (5), 469–477. 10.1007/s10570-005-4434-7.

[ref17] Piqué N.; Gómez-Guillén M.; Montero M. Xyloglucan, a Plant Polymer with Barrier Protective Properties over the Mucous Membranes: An Overview. Int. J. Mol. Sci. 2018, 19 (3), 673 10.3390/ijms19030673. 29495535 PMC5877534

[ref18] Burgalassi S.; Chetoni P.; Panichi L.; Boldrini E.; Saettone M. F. Xyloglucan as a novel vehicle for timolol: Pharmacokinetics and pressure lowering activity in rabbits. J. Ocul. Pharmacol. Ther. 2000, 16 (6), 497–509. 10.1089/jop.2000.16.497. 11132897

[ref19] Periasamy S.; Lin C. H.; Nagarajan B.; Sankaranarayanan N. V.; Desai U. R.; Liu M. Y. Mucoadhesive role of tamarind xyloglucan on inflammation attenuates ulcerative colitis. J. Funct. Foods 2018, 47, 1–10. 10.1016/j.jff.2018.05.035. 30555535 PMC6289526

[ref20] Silva B.; São Braz B.; Delgado E.; Gonçalves L. Colloidal nanosystems with mucoadhesive properties designed for ocular topical delivery. Int. J. Pharm. 2021, 606, 120873 10.1016/j.ijpharm.2021.120873. 34246741

[ref21] Nitta Y.; Fang Y.; Takemasa M.; Nishinari K. Gelation of xyloglucan by addition of epigallocatechin gallate as studied by rheology and differential scanning calorimetry. Biomacromolecules 2004, 5 (4), 1206–1213. 10.1021/bm034526y. 15244432

[ref22] Yan Y.; Takemasa M.; Zhao C.; Yu L.; Nishinari K. Structure-gelation research on gallate analogs and xyloglucan by rheology, thermal analysis and NMR. Food Hydrocolloids 2016, 52, 447–459. 10.1016/j.foodhyd.2015.07.012.

[ref23] Opare Kennedy D.; Kojima A.; Hasuma T.; Yano Y.; Otani S.; Matsui-Yuasa I. Growth inhibitory effect of green tea extract and (−)-epigallocatechin in Ehrlich ascites tumor cells involves a cellular thiol-dependent activation of mitogenic-activated protein kinases. Chem.-Biol. Interact. 2001, 134 (2), 113–133. 10.1016/S0009-2797(00)00251-9. 11311209

[ref24] Chan M. M.; Fong D.; Ho C. T.; Huang H. I. Inhibition of inducible nitric oxide synthase gene expression and enzyme activity by epigallocatechin gallate, a natural product from green tea. Biochem. Pharmacol. 1997, 54 (12), 1281–1286. 10.1016/S0006-2952(97)00504-2. 9393670

[ref25] Shao W.; Chen R.; Lin G.; Ran K.; Zhang Y.; Yang J.; Pan H.; Shangguan J.; Zhao Y.; Xu H. In situ mucoadhesive hydrogel capturing tripeptide KPV: The anti-inflammatory, antibacterial and repairing effect on chemotherapy-induced oral mucositis. Biomater. Sci. 2021, 10 (1), 227–242. 10.1039/D1BM01466H. 34846053

[ref26] Zhu W.; Mei H.; Jia L.; Zhao H.; Li X.; Meng X.; Zhao X.; Xing L.; Yu J. Epigallocatechin-3-gallate mouthwash protects mucosa from radiation-induced mucositis in head and neck cancer patients: A prospective, non-randomised, phase 1 trial. Invest. New Drugs 2020, 38 (4), 1129–1136. 10.1007/s10637-019-00871-8. 31701429

[ref27] Racaniello G. F.; Pistone M.; Meazzini C.; Lopedota A.; Arduino I.; Rizzi R.; Lopalco A.; Musazzi U. M.; Cilurzo F.; Denora N. 3D printed mucoadhesive orodispersible films manufactured by direct powder extrusion for personalized clobetasol propionate based paediatric therapies. Int. J. Pharm. 2023, 643, 123214 10.1016/j.ijpharm.2023.123214. 37423374

[ref28] Takeuchi K.; Watanabe M.; Yanagi M.; Murakami I.; Hosono H.; Nishizawa S.; Chigono Y.; Hirabayashi S.; Matsuda J.; Yamaoka K.; et al. In-vitro and clinical evaluation of an oral mucosal adhesive film containing indomethacin. Yakugaku Zasshi 2008, 128 (12), 1791–1795. 10.1248/yakushi.128.1791. 19043298

[ref29] Xing L.; Zhang H.; Qi R.; Tsao R.; Mine Y. Recent Advances in the Understanding of the Health Benefits and Molecular Mechanisms Associated with Green Tea Polyphenols. J. Agric. Food Chem. 2019, 67 (4), 1029–1043. 10.1021/acs.jafc.8b06146. 30653316

[ref30] TEIJIN limited. Interview form; Saliveht®Aerosol: Japan, 2023 p 4.

[ref31] Pestana A. M.; Calixto G. M. F.; Bezerra A. A. C.; de Morais Ribeiro L. N.; da Costa A. C.; Moraes Â. M.; Franz-Montan M. Analysis of Key Factors for Evaluating Mucosal Adhesion Using Swine Buccal Tissue. J. Pharm. Sci. 2024, 113 (8), 2413–2419. 10.1016/j.xphs.2024.04.018. 38657756

[ref32] Pham Q. D.; Nöjd S.; Edman M.; Lindell K.; Topgaard D.; Wahlgren M. Mucoadhesion: Mucin-polymer molecular interactions. Int. J. Pharm. 2021, 610, 121245 10.1016/j.ijpharm.2021.121245. 34755651

[ref33] Nep E.; Conway B. Grewia Gum 2: Mucoadhesive Properties of Compacts and Gels. Trop. J. Pharm. Res. 2011, 10 (4), 393–400. 10.4314/tjpr.v10i4.4.

[ref34] Fangueiro J. F.; Parra A.; Silva A. M.; Egea M. A.; Souto E. B.; Garcia M. L.; Calpena A. C. Validation of a high performance liquid chromatography method for the stabilization of epigallocatechin gallate. Int. J. Pharm. 2014, 475 (1–2), 181–190. 10.1016/j.ijpharm.2014.08.053. 25175728

[ref35] Li N.; Taylor L. S.; Mauer L. J. Degradation kinetics of catechins in green tea powder: Effects of temperature and relative humidity. J. Agric. Food Chem. 2011, 59 (11), 6082–6090. 10.1021/jf200203n. 21495730

