# Supplementary material for: Preparing and Characterizing of Xyloglucan Films Containing Tea Extract for Oral Mucositis
Source: ACS Omega. 2024 Dec 19;10(1):390–9. doi: 10.1021/acsomega.4c06410 (PMC11740623; doi:10.1021/acsomega.4c06410)
Supplement: Supplementary file 1 — ao4c06410_si_001.pdf [file ao4c06410_si_001.pdf]

Preparing and Characterizing of Xyloglucan Films Containing Tea Extract for Oral Mucositis

Kaoru Hirose [a‡], *Rieko Nitto* [a‡], *Shohtaro Yokota*[a], *Yayoi Kawano*[a*], *Kazuhiko Yamatoya*[b],

Akira Tabuchi[b], *Yumewo Suzuki*[b], *Takehisa Hanawa*[a**]

[a]Faculty of Pharmaceutical Sciences, Tokyo University of Science, Noda, Chiba, 2788510, Japan

[b]MP Gokyo Food & Chemical Co., Ltd., Kita-ku, Osaka, 5300001, Japan.

**Corresponding, Email: t-hanawa@rs.tus.ac.jp

‡ K.H. and R.N. contributed equally to this paper

Keywords

Hydrogel, Xerogel, Xyloglucan, Tea extract, Stomatitis, Film

*Present address: Graduate School of Pharmaceutical Sciences, Nagoya City University, 3-1

Tanabe-dori, Mizuho-ku, Nagoya 467-8603, Japan

Supporting Information: The results of statistical analysis in rupture strength and adhesion force

*:P<0.05, **P<0.01, n.s.: no significant

Table S1. Statistical analysis in rupture strength for Xylo/TE hydrogel film

Sample (Xylo/TE)	1.5/0.1	1.5/0.5	1.5/1.0	3.0/0.1	3.0/0.5	3.0/1.0
1.5/0.1						
1.5/0.5	**					
1.5/1.0	n.s.	*				
3.0/0.1	n.s.	**	n.s.			
3.0/0.5	**	n.s.	**	**		
3.0/1.0	**	**	**	**	**	

*:P<0.05, **P<0.01, n.s.: no significant

Table S2. Statistical analysis in rupture strength for Xylo/TE xerogel film

Sample (Xylo/TE)	1.5/0.1	1.5/0.5	1.5/1.0	3.0/0.1	3.0/0.5	3.0/1.0
1.5/0.1						
1.5/0.5	**					
1.5/1.0	**	n.s.				
3.0/0.1	**	**	**			
3.0/0.5	**	**	**	n.s.		
3.0/1.0	**	n.s.	**	**	**	

*:P<0.05, **P<0.01, n.s.: no significant

Table S3. Statistical analysis in rupture strength for Xylo/TE xerogel film Xylo/TE xerogel films swollen in water for 20 min

Sample (Xylo/TE)	1.5/0.1	1.5/0.5	1.5/1.0	3.0/0.1	3.0/0.5	3.0/1.0
1.5/0.1						
1.5/0.5	**					
1.5/1.0	n.s.	*				
3.0/0.1	n.s.	**	n.s.			
3.0/0.5	**	n.s.	**	**		
3.0/1.0	**	**	**	**	**	

*:P<0.05, **P<0.01, n.s.: no significant

Table S4. Statistical analysis of the water absorption behavior of Xylo/TE xerogel films.

Sample (Xylo/TE)	1.5/0.1	1.5/0.5	1.5/1.0	3.0/0.1	3.0/0.5	3.0/1.0
1.5/0.1						
1.5/0.5	**					
1.5/1.0	**	n.s.				
3.0/0.1	**	n.s.	n.s.			
3.0/0.5	**	n.s.	n.s.	n.s.		
3.0/1.0	**	**	**	**	**	

*:P<0.05, **P<0.01, n.s.: no significant

The data are collected 30 minutes after absorbing them.

Sample (Xylo/TE)	1.5/0.1	1.5/0.5	1.5/1.0	3.0/0.1	3.0/0.5	3.0/1.0	OTC No.1	OTC No.2	OTC No.3
1.5/0.1									
1.5/0.5	n.s.								
1.5/1.0	**	n.s.							
3.0/0.1	**	n.s.	n.s.						
3.0/0.5	**	**	n.s.	n.s.					
3.0/1.0	**	**	**	**	n.s.				
OTC No.1	n.s.	**	**	n.s.	n.s.	**			
OTC No.2	n.s.	**	*	*	n.s.	n.s.	**		
OTC No.3		**	**	**	**	n.s.	**	*	

Table S5. Statistical analysis in adhesion force for Xylo/TE xerogel films and OTC products.

*:P<0.05, **P<0.01, n.s.: no significant
